# Lack of action–sentence compatibility effect in non-clinical individuals with high autistic traits

**DOI:** 10.3389/fpsyg.2023.1293405

**Published:** 2023-12-06

**Authors:** Keisuke Irie, Shuo Zhao, Rieko Aruga, Amiri Matsumoto, Akari Ogawa, Nan Liang

**Affiliations:** ^1^Department of Advanced Occupational Therapy, Human Health Sciences, Graduate School of Medicine, Kyoto University, Kyoto, Japan; ^2^School of Psychology, Shenzhen University, Shenzhen, Guangdong, China

**Keywords:** autism spectrum quotient, action-sentence compatibility effect, low autistic traits, high autistic traits, mental rotation (MR)

## Abstract

**Introduction:**

Patients with autism spectrum disorder (ASD) exhibit atypical responses to language use and comprehension. Recently, various degrees of primary autistic symptoms have been reported in the general population. We focused on autistic traits and examined the differences in mechanisms related to language comprehension using the action–sentence compatibility effect (ACE). ACE is a phenomenon in which response is facilitated when the action matches the behavior described in the statement.

**Methods:**

In total, 70 non-clinical individuals were divided into low autistic and high autistic groups according to their autism spectrum quotient (AQ) scores. ACEs with adverbs and onomatopoeias were examined using a stimulus set of movement-related sentences. A choice-response task helped determine the correct sentence using antonym adverbs (slow and fast) and onomatopoeia (quick and satto) related to the speed of the movement.

**Results:**

The low-AQ group showed ACEs that modulated the reaction time in antonym sentences. The high-AQ group showed less temporal modulation, and their overall reaction time was shorter. The low-AQ group showed faster reaction times for onomatopoeic words; however, the high-AQ group showed a tendency to reverse this trend. In individuals with intermediate autistic traits, the angle effect may be moderated by individual differences in motor skills and experience rather than autistic traits. The stimulus presentation involved a passive paradigm.

**Discussion:**

This study provides insight into language comprehension processes in non-clinical individuals ranging from low to high autistic idiosyncrasy and elucidates language and behavior in individuals at different locations on the autistic trait continuum.

## 1 Introduction

Autism spectrum disorders (ASD) are syndromes characterized by qualitative impairments in interpersonal interactions and responses and a range of restricted behaviors and interests, as defined in the Diagnostic and Statistical Manual of Mental Disorders, fifth edition (DSM-5) Guide to the Classification and Diagnosis of Mental Disorders (American Psychiatric Association, 2013). The core disorder of ASD is the impaired capacity for social intuition in interpersonal relationships. Such social issues are mainly related to executive functions and communication—the skills needed to effectively perform a series of activities with a purpose. It includes temporarily storing information and controlling thoughts and actions (Miyake et al., 2000; Zelazo et al., 2004; Kaushanskaya et al., 2017). Moreover, it significantly affects many aspects of daily life and is associated with an individual's ability to communicate (Gilotty et al., 2002; Gruber and Goschke, 2004; Akbar et al., 2013). Communication problems reported in ASD patients include poor eye contact and joint attention (Mundy et al., 1986, 2009), inability to identify facial expressions (Weigelt et al., 2012; Tang et al., 2015), and non-verbal or verbal issues (Wetherby and Prutting, 1984). Additionally, various reports suggest that language mediates executive functions or that executive functions are the basis of communicative competence (Marcovitch and Zelazo, 2009; Long et al., 2018). Having problems understanding the instructions and intentions of others prior to execution is a core symptom of ASD. Further, language ability directly or indirectly predicts social adjustment in adults with ASD (Otsuka et al., 2017), and further research on language ability in individuals with ASD is needed to facilitate social adjustment.

Pragmatics is a field that focuses on the use of language in certain sociocultural situations, which is a component of social communication and includes many skills (McEvoy et al., 1993; Capps et al., 1998). Although children with ASD can acquire words, such as nouns, and correct syntax, they have pragmatic problems such as difficulties in understanding and using abstract expressions, both figurative and emotional as well as understanding the literal meanings of words (Happé, 1993; Minshew et al., 1997; Just et al., 2004). Other studies focusing on syntactic difficulties among children and adolescents with ASD have reported the omission or misuse of morphemes such as “-ed” in the past tense and “-s” in the third-person singular present tense (Eigsti et al., 2007; Park et al., 2012; Ambridge et al., 2015; Modyanova et al., 2017). Studies have also investigated the relationship between these executive functions and language (Haebig et al., 2015; Ellis Weismer et al., 2018). Although it is expected to influence social communication, such as directing behavior using language, the relationship between language understanding and physical performance has not been adequately tested. Several studies have suggested that ASD features are also present in non-clinical individuals to varying degrees (Chen and Yoon, 2011; Nummenmaa et al., 2012), suggesting that ASD can range from healthy at one extreme of the continuum to disability at the other (Baron-Cohen et al., 2001; Constantino and Todd, 2003). A study of non-clinical individuals used the autism spectrum quotient (AQ) questionnaire to measure the degree of autism in the population. Reportedly, typically developing individuals with high AQ have similar characteristics to those with ASD on joint attention tasks (Zhao et al., 2015). Thus, looking at associations among typically developing individuals is crucial for understanding phenomena occurring in our daily lives.

Over the past two decades, cognitive processing, including language comprehension, has become more than the mere manipulation of representations in the brain. This is called grounded cognition (Barsalou, 2008), in which sensorimotor areas of the cerebral cortex activate the same areas that are activated while recognizing and acting on verbal representations of concepts (Hauk et al., 2004; Aziz-Zadeh et al., 2006). Behavioral experiments have repeatedly reported that when judging a behavioral statement, the actions included in that statement are promoted (Glenberg and Kaschak, 2002; Glenberg et al., 2008). For example, Glenberg and Kaschak (2002) asked participants to judge the sensibility of sentences describing movements toward the reader (e.g., opening a door) and away from the reader (e.g., closing a door). Results showed that participants responded faster when body movements in the same direction as described in the sentence were mentioned than when movements in the opposite direction were mentioned. This action effect is an action–sentence compatibility effect (ACE; Diefenbach et al., 2013), and studies using various languages have confirmed that ACE can occur at the level of the intended action effect (Borreggine and Kaschak, 2006; Boulenger et al., 2006; Awazu, 2011). Bergen and Wheeler (2005) confirmed that this facilitation is observed not only in the direction of body movement but also with specific hand movements (e.g., clenching a chair or opening a palm). These studies suggest that understanding sentences associated with an action activates motor simulation, which accelerates the response. We tested ACEs when modifying verbs with the antonyms fast–slow, an adverb related to speed, and quick–satto (onomatopoeic), a synonym for onomatopoeia, in typically developing participants. Consequently, it promoted more behavioral responses to hand-related words than slow and, overall, satto promoted more behavioral responses than quick (Irie et al., 2021). Specifically, “satto” (e.g., open the door *satto*) promoted more responses than “quick” (e.g., open the door *quickly*).

Onomatopoeia is a general term for words that imitate sounds or describe the state of things and are frequently used in everyday speech as they directly express a perceived stimulus. Compared to words, such as nouns and verbs, in which the relationship between sound and meaning is arbitrary, onomatopoeia is a word whose sound and meaning are similar (Assaneo et al., 2011). Osaka reported that not only auditory regions but also the lingual gyrus, a higher visual area involved in processing facial expressions, and brain regions, such as the supplementary motor area and premotor area involved in preparing for the generation of a laughing face, were activated when participants were exposed to onomatopoeia related to laughter in a closed-eye condition (Osaka et al., 2003). Thus, simulation of word-related movements can help understand verbs and onomatopoeia and share the speaker's intended content. However, children and adolescents with ASD exhibit problems with motor imagery (MI) skills in mental rotation (MR) tasks (Conson et al., 2013; Chen et al., 2018). If MI is involved in the comprehension of words that describe actions, it is expected that individuals with ASD who exhibit problems with MI ability would be less likely to produce ACEs. Further, they would have great difficulty understanding abstract words, such as onomatopoeia. However, there are no studies on motor facilitation effects associated with autistic traits and word comprehension in non-clinical individuals. We used the AQ to investigate the differences in the mechanisms of language comprehension between people with high and low autistic traits using the ACE. We hypothesized that individuals with high AQ would be less likely to produce ACEs, that differences between fast–slow and quick–satto would disappear, and that there would be a positive correlation between AQ and MI ability.

## 2 Methods

### 2.1 Ethics statement

Participants were recruited through a bulletin board at the university, and informed consent was obtained after explaining the content of this study. The experimental procedures and protocols were performed in accordance with the Declaration of Helsinki and approved by the Ethics Committee of Kyoto University Graduate School and the Faculty of Medicine (approval number R2188-3).

### 2.2 Participants

An *a priori* analysis in the analysis of variance (ANOVA) using G^*^Power with effect size *f* = 0.25, α = 0.01, and power (1-β) = 0.95 was calculated for 68 participants. In this experiment, 70 participants (mean age = 21.4 ± 1.22 years, 23 women and 47 men) were enrolled. Exclusion criteria were (1) individuals whose native language was not Japanese and (2) inability to sufficiently respond to button presses because of cerebrovascular or orthopedic disease.

### 2.3 Apparatus

Stimulus presentation and data acquisition were conducted using E-Prime 3.0 (Psychology Software Tools, Inc.) on a personal computer (Windows 10 or 11). First, the participant sat on a chair approximately 60 cm away from the computer screen (21.5-inch display monitor) and placed their index finger of the right hand at a point marked 20 cm away from the response button. The computer screen then showed either a random sentence related to the action or a completely unrelated sentence. Participants were instructed to read the text presented and press the button only if they understood the text correctly.

### 2.4 Stimuli

Twenty sentences were prepared to describe actions related to hands and other body parts. The sentences were then transformed using the word “fast,” which is a word that modifies the speed of action and its antonym “slow” (e.g., “eat bread faster,” “walk faster”). The uses of the word “slow” include “eat bread slowly” and “walk slowly.” Thus, 40 sentences were created using the words “hand × fast” and “hand × slow,” and 40 sentences using the words “other × fast” and “other × slow.” In addition to these 80 sentences, 20 pseudo-sentences were prepared in which the noun-verb combination did not make sense (e.g., “pour hands”). One-hundred different sentences were prepared as stimulus Set 1. Stimulus Set 2 examined the usefulness of onomatopoeia using the onomatopoeia “satto,” which is synonymous with “quick.” One-hundred different sentences were prepared using the same procedure as that followed in stimulus Set 1. The validity of the stimuli and methods used in this experiment was confirmed by Irie et al. (2021).

### 2.5 Design

The experiment was constructed as a three-factor mixed randomized design with verb (Set 1: fast, slow, Set 2: satto, quick), body (hand, other), and group (high AQ, low AQ) as factors. Participants performed all tasks.

### 2.6 Measures

The AQ comprises 50 questions with sub-items on social skills, communication, imagination, and attention as areas of interest. Each of them was rated on a 10-item self-rating scale, and participants were asked to respond on a four-point scale with “strongly agree,” “somewhat agree,” “somewhat disagree,” or “strongly disagree.” Participants with higher AQ scores exhibited more autistic traits than those with lower AQ scores. AQ scores had good reliability and have been shown to have adequate sensitivity and specificity (Baron-Cohen et al., 2001). We used the median as a cutoff, as in previous studies (Zhao et al., 2015), to investigate characteristics in typically developing individuals as a preliminary step to a clinical sample. Participants with AQ scores lower than the median of 22.5 were assigned to the low-AQ group (M = 17, SD = 4.36, range = 6–22, *n* = 35), and those with higher scores to the high-AQ group (M = 26, SD = 4.58, range = 23–42, *n* = 35). There was no significant difference in sex ratio between the two groups (24 men and 11 women in the low-AQ group and 12 women and 23 men in the high-AQ group; Fisher's exact test, *p* > 0.1). In addition, language skills were tested with the Japanese Adult Reading Test and motor skills were tested with a choice-response task, which showed no correlation with the AQ (*p* > 0.1).

### 2.7 Procedure

The MI ability was evaluated using MR before the language task. Images of the left and right palms and the back of the hand were prepared, and 24 images were created by rotating them every 60°. The image was presented for 3,000 ms, and participants were requested to discriminate between the left and right hands as quickly and accurately as possible. The task was controlled using E-Prime, and the reaction time was determined.

**Figure 1:**
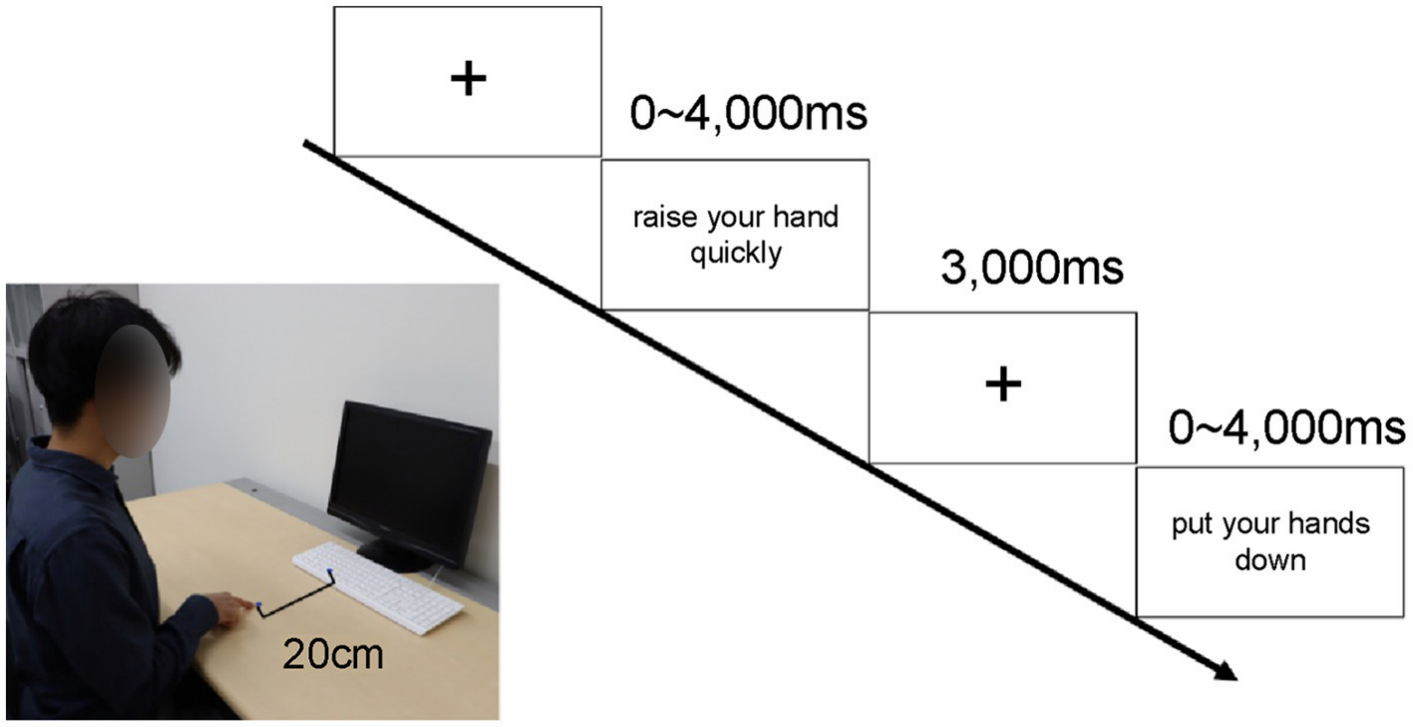
Experimental setup and protocol. Procedure: Each participant sat 60 cm away from the computer screen and placed the index finger of their right hand 20 cm away from the space key. The gazing point was displayed for 3 sec, and a sentence related to the action was presented for up to 4,000 ms. The participant responded by pressing the space key only if they understood the sentence. If the text was not understood, no response was provided, and after 4,000 ms, the next gazing point was displayed. Figure adapted from Irie et al., 2021, CC BY-4.0.

Participants practiced after being briefed on the experimental procedure (Figure 1). The test consisted of two blocks of 100 trials each—stimulus Set 1 and stimulus Set 2—and the order of trials was randomized by block sensitivity. In each trial, a cross was first presented in the center of the screen for 3,000 ms. Subsequently, a sentence or pseudo-sentence related to hand or other body part movements was presented for up to 4,000 ms. In each trial, participants were asked to judge whether the stimulus sentence made sense. Participants were instructed to press a button 20 cm away with their index finger only if they understood the sentence.

### 2.8 Analysis

If the sentence was not read, the reaction time was fast; conversely, the reaction time could be slow for reasons other than reading. Therefore, trials with reaction times of < 300 ms and trials with reaction times of more than three standard deviations were excluded before analysis to avoid the possibility of including false responses. The exclusion rate was 1.2%. The main analyses are repeated ANOVA for reaction time (RT) and the number of errors (NoE) for three factors: AQ (low-AQ group, high-AQ group), words (Set 1: slow–fast, Set 2: quick–satto), and body part (hand-related, other body-related conditions). If a two-way or three-way interaction was significant, a *post-hoc* (Bonferroni) test was performed (*p* < 0.05). In addition, comparisons of differences in MR angle between the low and high-AQ groups were made using two-way repeated measures ANOVA. Pearson's correlation analysis was then performed to determine if a relationship existed between mental MR and AQ. All statistical analyses were performed using IBM SPSS Statistics version 26 (IBM, Armonk, NY, USA).

## 3 Results

### 3.1 Influence of each factor alone or interactively on RTs

**Figure 2:**
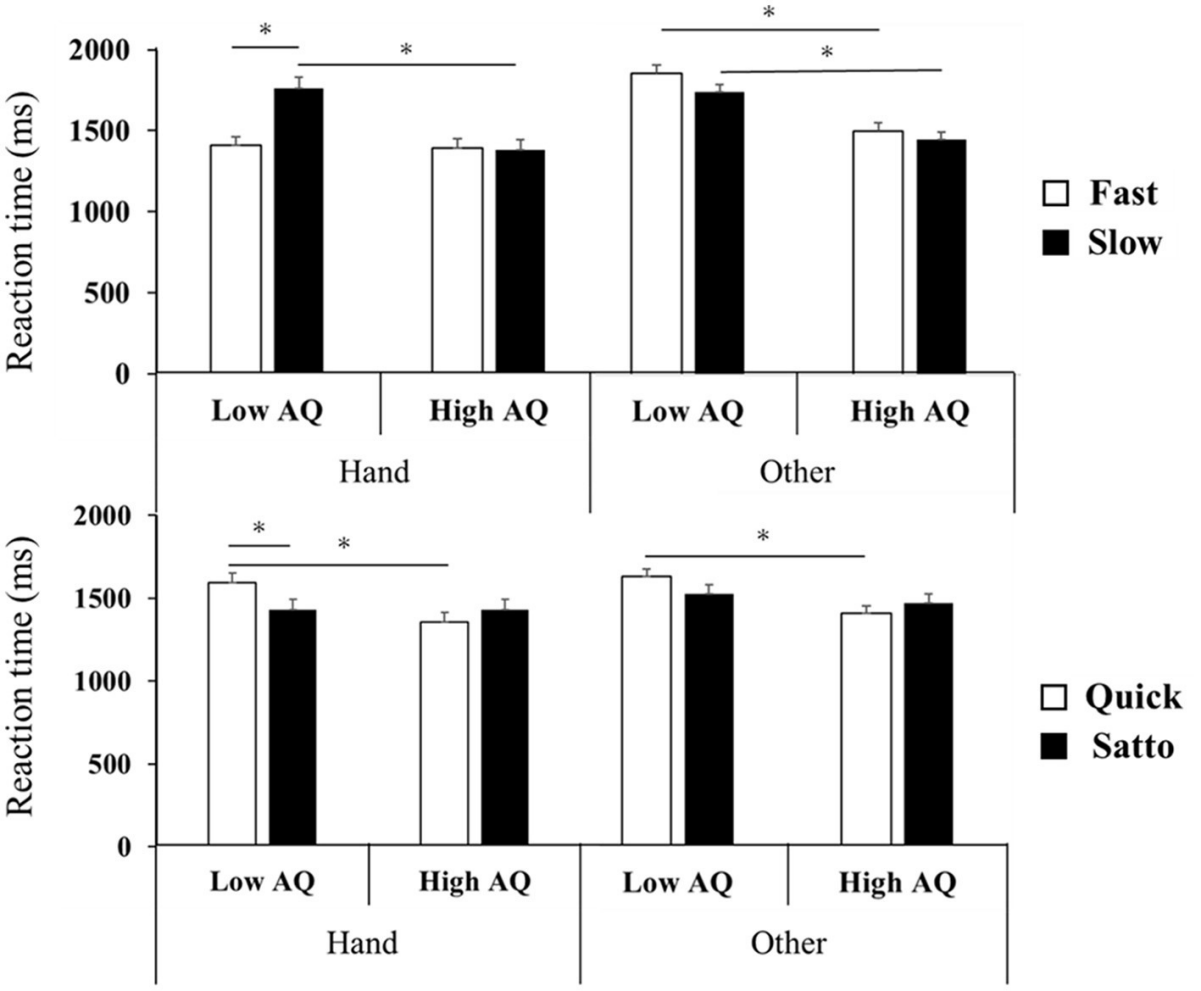
Results of experimental Set 1 **(top row)** and Set 2 **(bottom row)**. Set 1 showed an interaction between words and AQ, with fast having significantly shorter RTs than slow for hand-related words in the low-AQ group (*p* < 0.05). The high-AQ group had significantly shorter RTs than the low-AQ group for hand-related and other-site-related sentences (*p* < 0.05). Set 2 showed an interaction between words and AQ, with satto significantly shorter reaction time than quick in the low-AQ group (*p* < 0.05). The high-AQ group had significantly shorter RTs than the low-AQ group for hand-related and other-site-related sentences (*p* < 0.05). AQ, autism spectrum quotient; RT, reaction time. *P < 0.05.

In a repeated three-way ANOVA performed on the RTs in stimulus Set 1, the interaction effect (body parts, words, and AQ groups) was significant (*F*_(1, 34)_ = 31.4, *p* < 0.001, ηp^2^ = 0.48). Further, the interaction was significant for words and AQ (*F*_(1, 34)_ = 14.7, *p* = 0.001, ηp^2^ = 0.30). In the hand words condition, the *post-hoc* test indicated that in the low-AQ group did fast show significantly shorter RTs than slow. No significant differences were found in RTs for words other than hand, due to differences in language. Further, “slow and High AQ” showed significantly shorter RTs than “slow and Low AQ.” The high-AQ group showed significantly shorter RTs than the low-AQ group (*p* < 0.05, Figure 2).

Similarly, the interaction among body parts, words, and AQ groups was significant for stimulus Set 2. (*F*_(1, 34)_ = 5.8, *p* < 0.05, ηp^2^ = 0.15). Additionally, the interaction effect (words and AQ groups) was significant (*F*_(1, 34)_ = 72.2, *p* < 0.001, ηp^2^ = 0.68). In words regarding the hand, the *post-hoc* test indicated that in the low-AQ group was the RT significantly shorter than the word to which quick was assigned. No significant differences were found in RTs for words other than hand, due to differences in language. Further, “quick and high AQ” showed significantly shorter RTs than “quick and low AQ.” The condition “quick and high AQ” showed significantly shorter RTs than “quick and low AQ” (*p* < 0.05).

### 3.2 Influence of each factor alone or interactively on NoE

**Figure 3:**
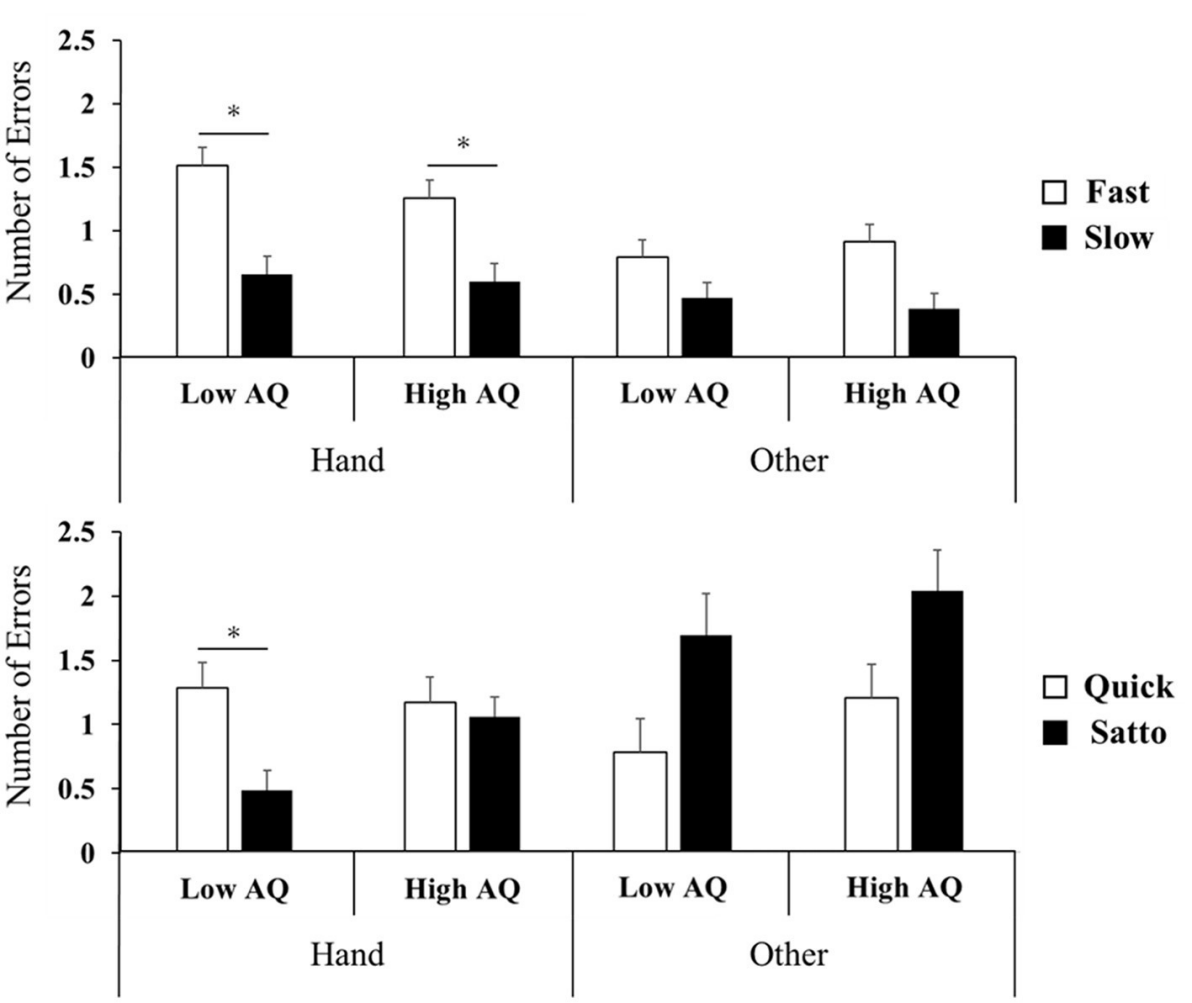
Number of error results for experimental Set 1 **(top row)** and Set 2 **(bottom row)**. Set 1 showed no interaction between words and AQ, indicating a main effect of words; fast had significantly more false responses than slow (*p* < 0.05). In Set 2, there was no interaction between the word and AQ, indicating the main effect of the word; in the low-AQ group, quick had significantly more false responses than satto (*p* < 0.05). AQ, autism spectrum quotient. *P < 0.05.

Concerning the number of false responses, a repeated three-way ANOVA on stimulus Set 1 did not confirm an interaction, indicating the main effect for words (*F*_(1, 34)_ = 44.8, *p* < 0.001, ηp^2^ = 0.57). *Post-hoc* testing showed that “fast” was associated with significantly more errors than “slow” in words related to hands (*p* < 0.05, Figure 3). For stimulus Set 2, the interaction effect between the body part and word was significant (*F*_(1, 34)_ = 25.6, *p* < 0.001, ηp^2^ = 0.43). In the *post-hoc* test, quick was significantly more NoE than satto for hand-related words only in the low-AQ group condition (*p* < 0.05, Figure 3).

### 3.3 Angular effects of MR and correlation of AQ and MR

**Figure 4:**
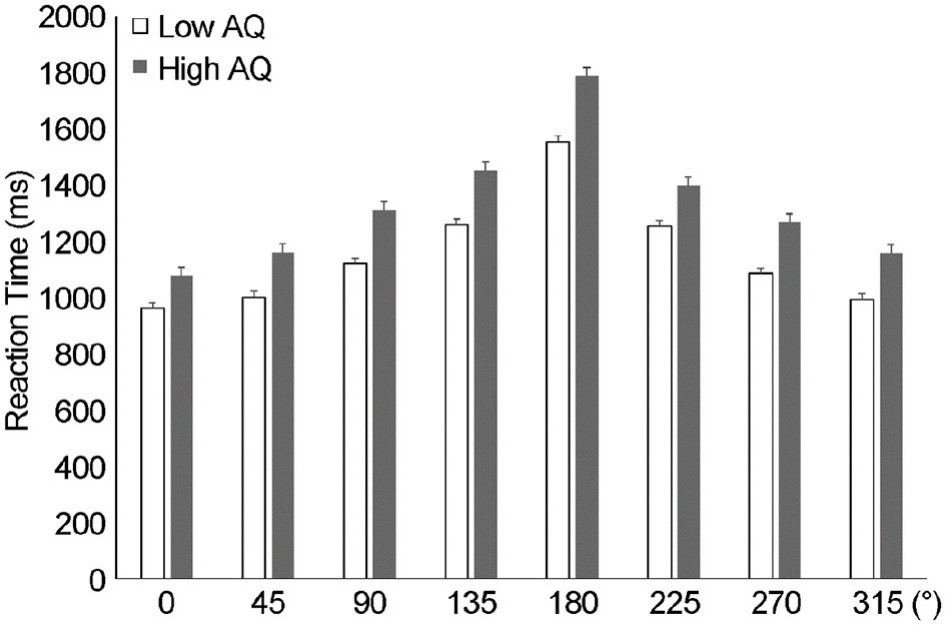
Reaction time results from the angle in mental rotation. The results of the reaction time according to the angle of rotation of the hand revealed that the reaction time was significantly slower at 180° (*p* < 0.001), indicating a change in reaction time according to the angle. AQ, autism spectrum quotient; RT, reaction time.

**Figure 5:**
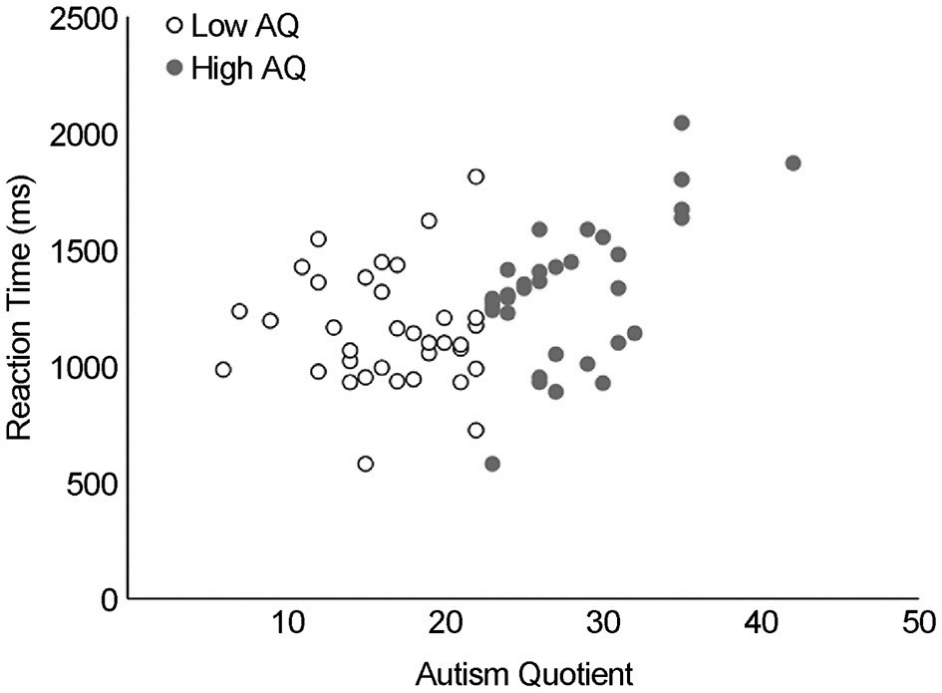
Correlation between autism quotient and MR reaction time. Pearson's product-moment correlation analysis revealed a significant correlation between the average reaction times of autism spectrum quotient and MR (*r* = 0.42, *p* < 0.01). MR, mental rotation.

As shown in Figure 4 for the rotational angle results, the change in RT peaked at 180° for both groups. Differences in MR RT between the two AQ groups (low and high AQ) and the angle of image rotation (0°, 45°, 90°, 135°, 180°, 225°, 270°, 315°) were examined using a two-way ANOVA (Figure 4). There was no interaction between AQ and rotation angle (*F*_(1, 34)_ = 0.82, *p* > 0.05, ηp^2^ = 0.024), with main effects for AQ (*F*_(1, 34)_ = 8.3, *p* < 0.01, ηp^2^ = 0.20) and rotation angle (*F*_(1, 34)_ = 103.3, *p* < 0.001, ηp^2^ = 0.75). *Post-hoc* tests showed significantly slower RTs in the high-AQ group than in the low-AQ group (*p* < 0.001). Pearson's product-moment correlation analysis revealed a significant correlation between the average RTs of AQ and MR (*r* = 0.42, *p* < 0.01, Figure 5).

## 4 Discussion

This study examined the effects of differences in autistic traits investigated in the AQ on ACEs produced by word comprehension in healthy non-clinical participants. Results showed an interaction between autistic traits and words. Specifically, in the low-AQ group, the adverb “fast” resulted in significantly shorter RTs than its antonym “slow,” and the onomatopoeic word “satto” resulted in significantly shorter RTs than “quick.” These results were similar to our previous study (Irie et al., 2021). However, there was no difference in these RTs in the high-AQ group. ACEs occurred more frequently with hand-related words in the low-AQ group and less frequently in the high-AQ group. Interestingly, the high-AQ group overall had shorter RTs than the low-AQ group. Further, a weak positive correlation was found between AQ and MR RT, and the mean RT for MR was significantly longer with higher AQ.

ACE is indicated by various neurophysiological evidence showing that motor and sensory systems are involved in the process of language comprehension (Buccino et al., 2005). Specifically, access to motor-related concepts is at least partially associated with the sensorimotor system via networks (Pulvermüller et al., 2005). The previous studies mentioned above are generally in agreement that language comprehension requires activation of brain regions related to the associated motor and sensory aspects to “simulate” the presented concept within the participant. However, there are challenges to its reproducibility, including ACE-related factors such as motor and spatial aspects. Further, there is an ongoing active debate about its (Goldinger et al., 2016; Mahon and Hickok, 2016; Greco, 2021; Winter et al., 2022). Nevertheless, the importance of motor simulation has been suggested as a feature of cognitive processing in individuals with ASD, with weaknesses in central integration (Frith and Happé, 1994) and overall A-style weakness that favors attention to detail over partial or global information processing (Happé, 1999; Klin et al., 2007; Tassini et al., 2022). This characteristic has been proposed to exhibit a normal distribution, with strengths varying from weak to strong in non-clinical participants (Moretti and Greco, 2018). Further, ASDs exhibit a normal distribution with central integration shifted toward the weak side. Based on the current results, it is inferred that ACEs occur in the low-AQ group because they simulate and understand whole sentences. However, ACEs are less common in the high-AQ group because they make judgments based on the partial understanding of sentences. The overall shorter RT of the high-AQ group than the low-AQ group can also be interpreted as above. A communication problem associated with the executive function of ASD individuals is the difficulty in capturing the intent of the speaker and listener. Frequently, they take “ambiguous expressions” overly literal (Happé, 1995). For example, if someone says, “We will be going swimming in the mini bus” or someone says, “cried his eyes out,” they become confused. For the first time, this study examines differences in autistic idiosyncrasies in typically developing individuals by means of ACE. Although only words related to movement were studied, the results suggest that individuals with highly autistic idiosyncrasies may have difficulty imagining the intent or movement of a sentence just by reading it.

Bonnet et al. (2022) reported that MI training improves language comprehension. The results suggest that motor training could strengthen the functional relationship between language and the motor system, leading to improved language comprehension and language production. It is well known that brain activity overlaps during MI actual action execution and action observation, suggesting that these different processes share similar movement-related representations (Jeannerod, 1994, 2001). MIs are generally classified as motor sensory MI (kMI) and visual MI (vMI), with kMI involving simulation processes that implicitly use motor sensory information, whereas vMI involves visuospatial restoration (Rodrigues et al., 2010; Grangeon et al., 2011). The kMI is studied using a presented hand rotation angle task. The existence of biomechanical effects or angles in which RT changes with increasing hand rotation angle indicates that the hand rotation task was performed using kMI rather than vMI (Parsons, 1994). The behavioral instructions asked participants to use kMI, and the results showed an angular effect, suggesting that many participants engaged in the task using kMI. A higher AQ was also associated with an increased mean RT in the MR task, supporting previous research. The lack of correlation between AQ and verbal and motor abilities among participants suggests that the ACE results were related to differences in kMI ability owing to autistic traits.

Prior research found a trade-off between the promotion of physical response and the number of false responses (Irie et al., 2021). Similarly, in this study, Set 1 confirmed a trade-off relationship between RT and NoE in the low-AQ group. However, in Set 2, “quick” yielded a slower RT and more NoE than “satto” in the low-AQ group. This could be because the language for “quick” does not correspond to the sentence, which could have changed the level of difficulty. Other possible effects of attention and impulsivity issues are also considered. Onomatopoeia is a symbolic verbal expression of bodily sensations, among which onomatopoeia and onomatopoeic words express aspects of external sounds and objects. They are also used from the earliest stages of development to associate words with sounds and evoke sound characteristics (Motamedi et al., 2021; Schlegel et al., 2021). Words based on bodily sensations, such as mimetic words related to actions, were used. In language comprehension, knowledge of words and contextual information from preceding words influences word comprehension (Stevenson et al., 1982; Hambrick, 2003). However, the information used to understand onomatopoeia is not limited to linguistic information–such as lexical knowledge–but also includes many other types of information, such as sensory information perceived by the individual (Osaka et al., 2003), as evidenced by the addition of a section on sensory issues in the DSM-5. Carne et al. investigated sensory processing in adults with ASD using the Sensory Profile and established that adults with ASD scored significantly higher than typically developing controls in the four quadrants of “low registration,” “sensory sensitivity,” and “sensory avoiding” (Crane et al., 2009). In addition, a study investigating the relationship between ASD characteristics and sensory processing in the general population found a high correlation between AQ and sensory scores (Robertson and Simmons, 2013). This suggests a potential relationship between autism traits and sensory processing problems in the general population. Differences in sensory traits because of autistic tendencies could affect the occurrence of ACEs and comprehension of onomatopoeia.

### 4.1 Limitations

First, since the AQ is a continuous index, the correlations found between the overall AQ score and MR RTs are of great interest. One possibility is that in individuals with intermediate rather than high or low autistic traits, the angle effect could be modulated by individual differences in motor skills and experience rather than autistic traits. Second, stimulus presentation consisted of a passive paradigm. Social interactions in real life are usually characterized by auditory-verbal-behavioral reciprocity and interdependence rather than by simple passive stimulus-response patterns. Given that verbal-behavioral interactions also occur in the auditory language (Kaschak et al., 2006), it could be beneficial in the future to investigate individuals' responses to verbal stimuli with more interactive response patterns in conjunction with the autistic trait spectrum. Furthermore, this result was obtained only from samples with high-functioning autistic traits, and cannot be generalized to ASD, including low-functioning individuals.

## 5 Conclusions

The group with low autistic traits was more likely to experience a facilitating effect on physical responses associated with word comprehension. However, this effect was not observed in those with high autistic traits. Interestingly, the results also indicated that the degree of onomatopoeia comprehension could differ between low and high autistic traits. These results suggest that sentence comprehension mechanisms could differ across autistic idiosyncrasies.

## Data availability statement

The raw data supporting the conclusions of this article will be made available by the authors, without undue reservation.

## Ethics statement

The study was approved by the Ethics Committee of Kyoto University Graduate School and the Faculty of Medicine (approval number R2188-3). The studies were conducted in accordance with the local legislation and institutional requirements. Written informed consent for participation in this study was provided by the participants' legal guardians/next of kin.

## Author contributions

KI: Writing – original draft. SZ: Writing – review & editing. RA: Writing – review & editing. AM: Writing – review & editing. AO: Writing – review & editing. NL: Methodology, Writing – review & editing.
